# Effects of low-dose aspirin on the osseointegration process in rats

**DOI:** 10.1186/s40729-020-00283-x

**Published:** 2021-01-13

**Authors:** Ana Carolina Lupepsa, Paula Vargas-Sanchez, Marcella Goetz Moro, Leomar Emanuel Almeida Mecca, Marcela Claudino, Priscilla Barbosa Ferreira Soares, Amanda Regina Fischborn, Jéssica Daniela Andreis, Gilson Cesar Nobre Franco

**Affiliations:** 1 Department of Health Sciences, State University of Ponta Grossa - UEPG, Ponta Grossa, PR Brazil; 2 Department of Dentistry, State University of Ponta Grossa - UEPG, Ponta Grossa, PR Brazil; 3 Department of Dental and Maxillofacial Surgery and Implantology, Federal University of Uberlandia - UFU, Uberlandia, MG Brazil; 4 Department of General Biology, Universidade Estadual de Ponta Grossa - UEPG, General Carlos Cavalcanti, 4748, Ponta Grossa, Paraná, 84030-900 Brazil

**Keywords:** Aspirin, Bone deposition, Dental implant

## Abstract

**Background:**

Several drugs are capable of promoting changes in bone metabolism. The aim of this study was to evaluate the effect of long-term low-dose aspirin (LDA) therapy on implant osseointegration.

**Methods:**

Male Wistar rats were divided into 4 groups (*n* = 8/group) according to oral gavage solution received prior (42 days) to the implant surgery on the tibia. The control group was treated with saline solution for 7 (CG-7) and 28 (CG-28) days. The use of low-dose aspirin was performed in AG groups (6.75 mg/kg of aspirin) for 7 (AG-7) and 28 (AG-28) days. After experimental periods, histomorphometric evaluation of bone-to-implant contact (BIC) and the bone area between threads (BABT) was performed.

**Results:**

Reduced BIC values were detected in AG-7 (62.8% ± 17.1) group compared to AG-28 (91.9% ± 5.4), CG-7 (82.7% ± 15.2), and CG-28 (89.9% ± 9.7). BABT evaluation revealed lower values in AG-7 (70.9% ± 15.2) compared to AG-28 (95.4% ± 3.7) and CG-28 (87.1% ± 10.2) groups.

**Conclusions:**

The treatment with low doses of aspirin promoted a discrete inhibitory effect in the early stages (7 days) of repair after implant placement, specifically in the bone deposition. However, these effects were not detected in the late stages (28 days), considering BIC and BABT parameters.

## Introduction

Dental implants have been considered as a safe and predictable strategy of rehabilitation for partially or completely edentulous patients with high success rates [1, 2]. These elevated success rates are essentially associated with osseointegration, which is defined as a requisite for the long-term success of implant-supported prostheses. However, bone metabolism may be influenced by several factors, resulting in alterations of the osseointegration process [3–5].

Several drugs are capable of promoting changes in bone metabolism [6]. In this context, the chronic use of non-steroidal anti-inflammatory drugs (NSAIDs) is described as possible modulators of bone metabolism since the cyclooxygenase enzyme (COX) plays a key role in osteoblastic and osteoclastic cell development, including the differentiation and activation processes [7]. Moreover, prostaglandin E2 (PGE2), produced by COX, is essential to vasodilation and angiogenesis that occurs during the early stages of new bone formation [7, 8].

Aspirin, also called acetylsalicylic acid, is known as a group of drugs that belongs to NSAIDs and inhibits cyclooxygenase-1 (COX-1) and COX-2 enzymes. The mechanism of action is based on the inhibition of prostaglandin synthesis through inactivation of COX; it exhibits analgesic, anti-inflammatory, and antipyretic effects. In the 1980s, the FDA approved the use of aspirin in low doses for the prevention of cardiovascular diseases. This clinical indication is related to the inhibition of platelet aggregation decurrent of the reduction of thromboxane synthesis, mediated by COX-1 in platelets [9–12]. Daily consumption of aspirin tablets is around 200 million worldwide, being considered one of the most used drugs in medical practice. In this context, clinical dental care for patients undergoing drug treatment for cardiovascular diseases has increased considerably today.

This biological effect is associated to lower doses (75–300 mg) when compared to its analgesic/anti-inflammatory/antipyretic doses (500–1000 mg) [13]. However, previous studies have demonstrated that the use of low-dose aspirin (LDA) is associated with increased bone mineral density (BMD) and reduced fracture risk [14, 15]. Contrarily, other authors have reported that low-dose aspirin is associated with an increased risk of fractures [16] and delayed bone healing [17]. And other authors also reported that long-term use of low-dose aspirin is not associated with lower BMD [18]. In addition, no clinically significant protective effect on the risk of fractures was detected by other authors [19].

Besides these controversial data, the biological impact of LDA on the osseointegration of dental implants remains poorly described. Moreover, the demand for patients under LDA treatment has increased significantly in the last years [20]. Therefore, the aim of this study was to evaluate the effect of long-term treatment of low-dose aspirin on the implant osseointegration using a rat model.

## Material and methods

Experimental protocol followed the ARRIVE Guidelines for Animal Research by the National Centre for the Replacement Refinement & Reduction [21] and was approved by a local Ethics Committee for Animal Experimentation (protocol # 041/2014). The sample size was calculated using the G*Power 3.112 software with type I (α) and type II (β) errors of 5% and 20% respectively. All animals were provided from the State University of Ponta Grossa (UEPG, Ponta Grossa/Paraná–Brazil). Aspirin was prepared daily by suspension with 0.5% sodium carboxymethylcellulose.

Thirty-two male *Wistar* rats, 10–12 weeks old, weighing between 400 and 450 g, were housed in plastic cages (4 animals/cage) under a 12h light/dark cycle at a temperature of 22 °C with food and water ad libitum. The animals were randomly divided into 4 groups (*n* = 8/group), according to oral gavage solution received prior (42 days) to the implant surgery on the tibia. Control groups (CG) were treated with saline solution for 7 (CG-7) and 28 (CG-28) days. The use of low-dose aspirin (6.75 mg/kg of aspirin) was performed in AG-7 and AG-28 for 7 and 28 days, respectively.

After 42 days of oral gavage with the respective treatment, implant placement was performed. For these procedures, the animals were submitted to an anesthetic procedure with a combination of intramuscular ketamine (90 mg/kg) and xylazine (10 mg/kg), after 8 h of a preoperative fasting period. Trichotomy followed by antisepsis with an iodopovidone solution was performed in the medial region of the tibia, and an incision of approximately 3 cm in length was performed in the right tibia. After dissection, the bone surface of the tibial metaphysis was exposed, and one conventional titanium implant (1.5 × 8.0 mm) with machined surfaced was placed. Implants were placed using a progressive sequence of drills under saline cooling, according to the manufacturer’s instructions (Neodent, Curitiba, Parana, Brazil). The soft tissues were sutured in separate layers with absorbable wire 5.0 (Vicryl Ethicon, Johnson & Johnson, São Paulo, São Paulo, Brazil) and externally with silk thread 4.0 (Vicryl Ethicon; Johnson & Johnson, São Paulo, São Paulo, Brazil).

A single dose of streptomycin-associated penicillin (0.1 mL/kg) (Vital Farma, Porto Alegre, Rio Grande do Sul, Brazil) and sodium dipyrone (20 mg/kg) (Ibasa, Porto Alegre, Rio Grande do Sul, Brazil) was administered immediately after the surgery. One single trained operator performed all surgical procedures.

The animals were euthanized by an overdose of anesthesia on the seventh and twenty-eighth postoperative days. Tissue blocks containing the implant and tibial fragment were fixed in 10% buffered formalin solution for 24 h and washed in running water for 24 h. These samples were dehydrated through an ethanol gradient of 70%, 80%, 90%, and 100% with 7 days for each phase at 5 °C. Following dehydration, the samples were embedded in a methacrylate-based resin (LR White, Berkshire, UK) according to the manufacturer’s instructions.

Histological sections were obtained (300-μm thickness) using a precision diamond saw, reduced to a final thickness of approximately 40 μm by grinding and polishing and stained in 1% toluidine blue (Sigma-Aldrich, San Louis, Missouri, USA). All histological sections were identified with a random numerical sequence in order to codify experimental periods and groups. Histomorphometric evaluation was performed using an optical microscope (Axio Imager A1M, Carl Zeiss, Germany) attached to a digital camera (Axiocam ICc3, Carl Zeiss, Germany). The acquired digital images were analyzed by a single and calibrated examiner blind to experimental groups and periods. The osseointegration process was evaluated throughout measurements of bone-to-implant contact (BIC) and the mineralized bone area between threads (BABT) using the software ImageJ 1.4 software (Version 1.5a Wayne Rasband, Bethesda, MD, USA).

Initially, the results were submitted to the Shapiro-Wilk normality test. Considering parametric data, one-way ANOVA followed by post hoc Tukey test was carried out for the multiple comparisons among the groups. The significance level adjusted at 5% was used for all statistical analysis (Graphpad Software Inc, USA).

## Results

**Fig. 1:**
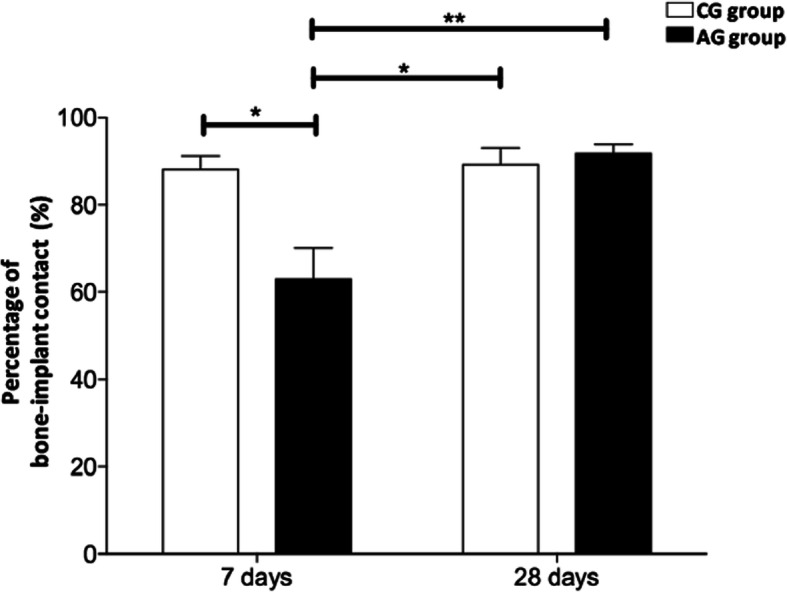
Effect of low-dose aspirin regarding bone-to-implant (BIC). The data represent mean ± SE (*n* = 8 animals/group). Asterisk indicates significant difference between the groups (*p < 0.01; **p < 0,001, ANOVA and post hoc Tukey)

The bone tissue present on the surface of the implants showed characteristics of vitality, evidenced by the presence of osteocytes inside the gaps. The bone deposition was assessed quantitatively with a two-dimensional evaluation: bone-implant contact (BIC) and the bone area between threads (BABT). After 7 days, a significant reduction in BIC values was detected in the presence of a low-dose aspirin (AG-7 group), compared to the control group (CG-7 group) (*p* < 0.05, Figs. 1 and 3). However, this reduction in BIC results was not detected between CG and AG groups after 28 days (*p* > 0.05, as illustrated in Figs. 1 and 3). Considering the different experimental periods in the same group, a significant increase in BIC values was observed in the AG-7 and AG-28 groups (*p* < 0.05, Fig. 1). There were no differences between CG-7 and CG-28 groups (*p* > 0.05, Fig. 1).

**Fig. 2:**
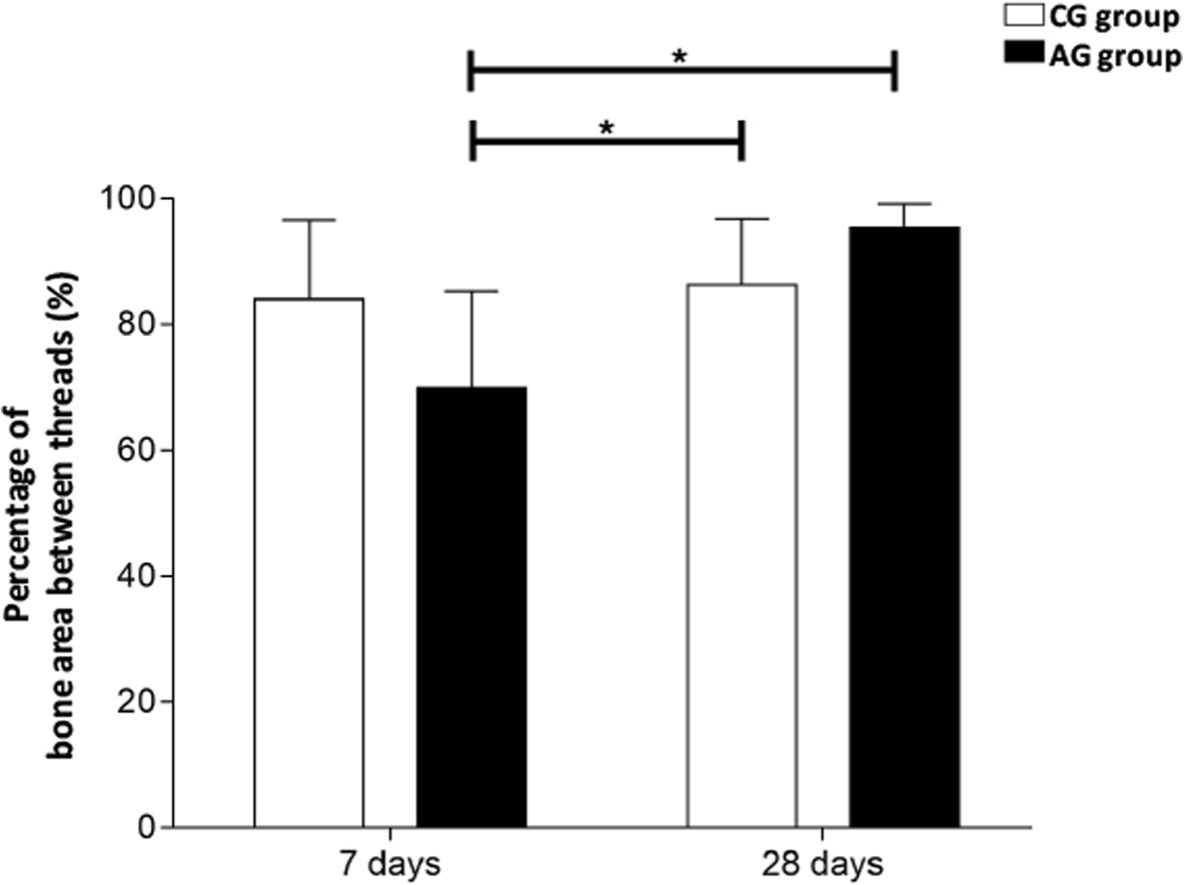
Effect of low-dose aspirin regarding bone area between threads (BABT). The data represent mean ± SE (*n* = 8 animals/group). Asterisk indicates significant difference between the groups (*p < 0.01, ANOVA and post hoc Tukey)

**Fig. 3:**
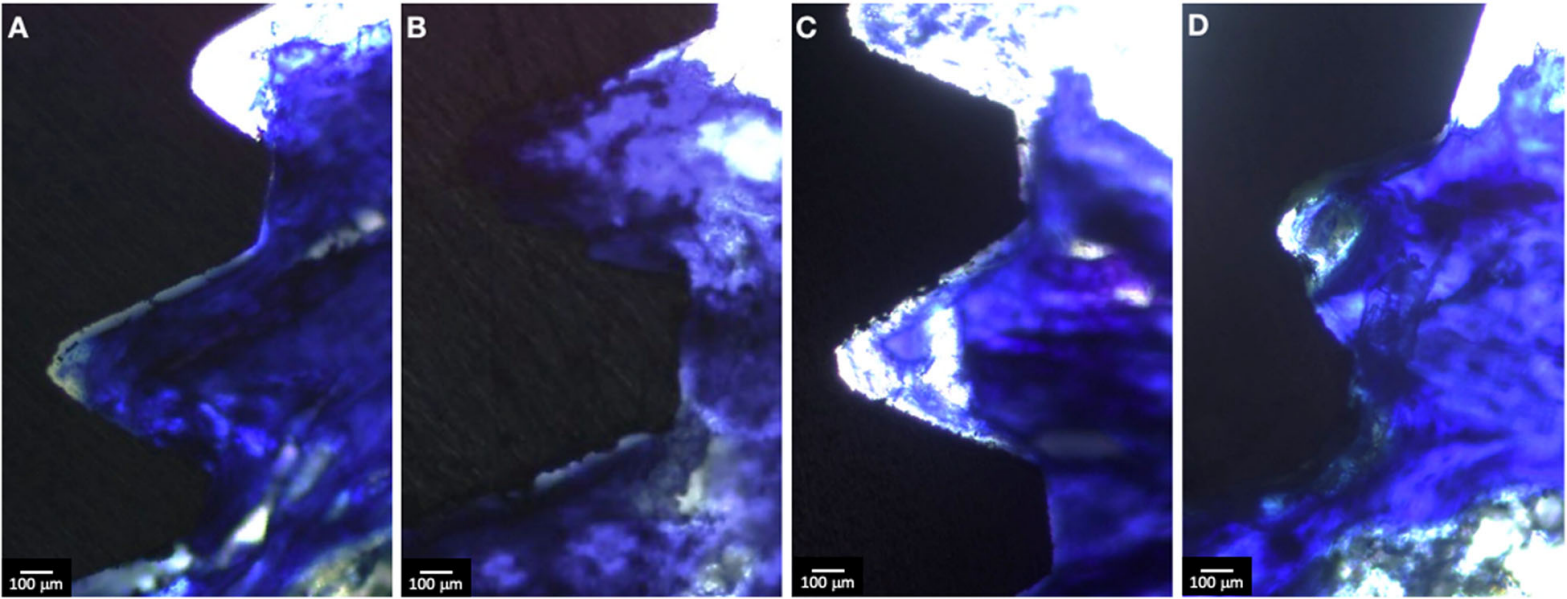
Histological findings of low-dose aspirin regarding bone deposition in titanium surface. Samples of AG-7 (**a**), AG-28 (**b**), CG-7 (**c**), and CG-28 (**d**) groups were embedded in a methacrylate-based resin and submitted to histological evaluation with toluidine blue staining

Following the pattern of BIC results, there was a trend towards and a reduction was observed in the AG-7 group, compared to CG-7 (*p* > 0.05, Figs. 2 and 3). However, no significant differences were observed between the groups CG-7 and AG-7, as well as CG-28 and AG-28 (*p* > 0.05, Figs. 2 and 3).

## Discussion

Several studies have reported high success rates of dental implants, which are strongly related to the osseointegration process [1, 2]. This process is characterized by bone deposition and resorption, involving mainly osteoblasts and osteoclasts [22]. Several systemic drugs can influence these biological events and may influence the clinical success of dental implants [23, 24]. Among these drugs, aspirin in low doses has been widely used for the prevention of cardiovascular diseases [25–27]. However, the putative effects of this long-term treatment on the implant osseointegration remain poorly described.

In our study, we evaluated bone deposition on the titanium implants placed in the tibia of rats treated with a low dose of aspirin. Our results showed a reduction in BIC values after 7 days of implant placement in the presence of treatment with LDA compared to the control group. However, this difference was not detected in the 28-day experimental period. Regarding the BABT data, only a tendency to decrease in the group treated with LDA was observed in relation to the control group after 7 days. As observed in BIC results, the BABT values of both groups after 28 days were higher when compared to the 7-day groups. Thus, these data suggest that treatment with LDA may delay the initial stages of bone repair.

In fact, it has been demonstrated that aspirin delayed bone healing with a threshold equivalent to a human dose of 325 mg using a rabbit ulnar osteotomy model [17]. Similar findings were also observed in rabbits, characterized by a significant decrease in bone growth after treatment of high-dose aspirin [28]. Also, it was demonstrated that the use of a non-selective COX inhibitor (Ketorolac) led to a delay in the fracture healing, but on the 35th day, all fractures (control and test groups) showed union [29]. Simon and collaborators [30] also demonstrated in an animal model of a bone repair study that NSAIDs can be a negative interference during the early stages of fracture union, leading to a reduction of fracture callus mechanical properties when administered during the initial stages of healing is performed.

Regarding the use of aspirin in low daily doses, it has been demonstrated that this therapy does not negatively impact bone density but could potentially increase bone resorption in diabetic mice [31]. Contrarily, other authors reported higher bone density and in ovariectomized mice treated with LDA [32] and increased bone deposition in the implant surface using the osteoporotic rat model [33].

In fact, it has been demonstrated that LDA suppressed osteoclast formation, osteoclastic-related gene expression, and osteoclastic bone erosion in a dose-dependent manner. Moreover, aspirin reduced osteoclast formation by suppressing receptor activator of nuclear factor kappa-B ligand-induced activation of extracellular signal-related kinase, p-38 mitogen-activated protein kinase, and c-Jun N-terminal kinase [34]. In addition, cyclooxygenase-inhibiting drugs have shown important reduction properties in the expression of “bone morphogenetic protein 2 (BMP-2),” which is related to osteoblastic differentiation and activation; (II) decrease in the “Core binding factor alpha 1 (CBFA-1)” transcription factor, which controls the production of osteocalcin, type 1 collagen, osteopontin, and bone sialoprotein; and (III) decreased initial vasodilation and expression of the “endothelial growth factor” (VEGF). All biological events are mediated by COX inhibition [35, 36]. However, these studies have been performed using longer experimental periods and different bone defects.

An important aspect of our study was the chosen dose (6.75 mg/kg), which is equivalent to approximately 75–80 mg in humans, the lower clinical dose used for cardiovascular protection. This dose was determined using the formula suggested by the Food and Drugs Administration (FDA) for dose conversion from animal to human studies [34, 35]. The dose of 75–80 mg is commonly used in medical practice since studies have shown that the side effects related to gastric lesions are directly associated with the dose, and thus, individuals exposed to higher cardioprotective doses (300 mg) have an increased risk of developing gastric damage and often complicating treatment adherence [37, 38].

In addition, treatment with low doses of aspirin promoted a discrete inhibitory effect in the early stages of tissue repair after implant placement, specifically in the bone deposition. However, these data must be strictly interpreted since they do not contraindicate the use of implants as a rehabilitation strategy for edentulous patients. In these cases, it is important to carefully evaluate the loading of these implants, especially in the early stages. However, some limitations may be observed in our study. Histological processing employed in our methodology does not result in semi-serial sections. In addition, the sections have a greater thickness and do not allow evaluations in some magnitudes.

Several studies have also been carried out to evaluate bone deposition in titanium implants submitted to different types of surface treatment [39–41]. Most of these studies demonstrate that surface treatment optimizes bone deposition. Thus, most companies provide only implants subjected to surface treatments, which can be considered a limitation of this study. In fact, our results show the effect of long-term low-dose aspirin therapy alone. However, it would be relevant for other studies to address the effect of surface treatment, associated with the use of low doses of aspirin. These studies will certainly contribute to the elucidation of the biological mechanisms involved in this process and will optimize the use of dental implants in the rehabilitation of edentulous patients submitted to long-term low-dose aspirin therapy.

In conclusion, the treatment with low doses of aspirin promoted a discrete inhibitory effect in the early stages (7 days) of repair after implant placement. However, these effects were not detected in the late stages (28 days).

## Data Availability

Not applicable.
